# The Relationship Between Femoral Head Avascular Necrosis and Erectile Dysfunction: A Retrospective Case–Control Study Conducted in Türkiye

**DOI:** 10.3390/jcm14082674

**Published:** 2025-04-14

**Authors:** Ekrem Özdemir, Ahmet Alper Özdeş, Fatih Emre Topsakal, Nasuhi Altay, Hüseyin Utku Özdeş, Esra Demirel

**Affiliations:** 1 Department of Orthopedics and Traumatology, Erzurum City Hospital, 25240 Erzurum, Türkiye; drfatihtopsakal@hotmail.com (F.E.T.); onasuhialtay@hotmail.com (N.A.); esrademirel82@gmail.com (E.D.); 2 Department of Urology, Karakoçan State Hospital, 23600 Elazığ, Türkiye; ahmetalperozdes@gmail.com; 3 Department of Orthopedics and Traumatology, İnönü University Faculty of Medicine, 44280 Malatya, Türkiye; dr.utkuozdes@gmail.com

**Keywords:** osteonecrosis, hip joint disease, sexual dysfunction, men’s health

## Abstract

**Background/Objectives:** Femoral head avascular necrosis (FAN) is a serious orthopedic disorder that causes the death of bone tissue as the outcome of the occlusion or insufficiency of the vessels supplying blood to the femoral head. It is especially common in middle-aged men. Factors such as alcohol consumption, corticosteroid use, trauma, and systemic diseases have influential roles in the development of FAN, and the ensuing vascular disruptions can also negatively affect the patient’s broader systemic vascular health. Erectile dysfunction (ED) is a condition caused by an impairment in penile blood flow, which reduces quality of life in men and has psychosocial effects. This study examined the potential relationship between FAN and ED in consideration of the similar pathophysiological mechanisms of these conditions. **Methods:** The research was planned as a retrospective case–control study and conducted between January 2020 and December 2023. FAN was diagnosed based on the Ficat–Arlet classification using plain radiography and magnetic resonance imaging, and staging was performed with expert clinical evaluations. The International Index of Erectile Function (IIEF) scale was administered to evaluate ED. Data from 50 patients with FAN and 50 healthy men were evaluated using appropriate statistical methods, including univariate comparisons and correlation analysis. **Results:** The analysis demonstrated a significant association between increasing FAN stages and greater severity of erectile dysfunction (ED). ED was identified in 35 out of 50 patients in the FAN group (70%), compared to 15 out of 50 individuals in the control group (30%). This difference was statistically significant (*p* < 0.05), indicating a substantially higher prevalence of ED in patients with FAN. A significant negative correlation was also observed between the FAN stage and International Index of Erectile Function (IIEF) scores (Spearman’s rho = −0.631; *p* = 0.001). The mean IIEF score was 23.4 in patients with FAN stage 1, which declined to 9.6 in those with stage 4, reflecting a marked deterioration in erectile function with advancing FAN stages. No statistically significant difference was found in the age distribution between the FAN and control groups (*p* > 0.05). **Conclusions:** This study demonstrates that, with the progression of FAN, systemic vascular deterioration affects penile blood flow, increasing the severity of ED. This finding highlights the common pathophysiological mechanisms of FAN and ED and reveals the importance of early diagnosis and multidisciplinary treatment approaches. This relationship should be examined in detail with larger samples and prospective designs in future studies.

## 1. Introduction

Femoral head avascular necrosis (FAN), particularly seen in middle-aged men, is a serious orthopedic condition involving the death of bone tissue as a result of the occlusion or insufficiency of the vessels supplying blood to the femoral head [1]. Various risk factors, such as corticosteroid use, alcohol consumption, trauma, and systemic diseases, have influential roles in the development of FAN. Cases of FAN are increasing over time, and the complications have devastating effects on quality of life. However, early diagnosis and accurate staging could change the course of the disease [2].

FAN is seen at varying rates around the world but its frequency increases in men in the high-risk group between the ages of 20 and 40. Studies have shown that the presence of the abovementioned risk factors significantly increases the incidence of the disease in comparison to individuals lacking these risk factors [3]. Furthermore, pathological differences are clearly observed between the early and advanced stages when FAN patients are evaluated with clinical staging. Staging is therefore an effective prognostic tool in the management of the disease and plays a key role in determining treatment strategies [4].

Erectile dysfunction (ED) is the most common sexual dysfunction in men and has negative effects on quality of life and psychosocial status [5]. The pathogenesis of ED arises from a combination of vascular, neurogenic, hormonal, and psychological factors and its prevalence increases with age. Approximately 20% of men over the age of 40 have symptoms of ED, but recent studies also show that the prevalence is rapidly increasing in young adults [6]. The majority of ED cases have a vascular basis and disorders of the vascular system are highly effective in its emergence.

We hypothesized a potential relationship between these two clinical conditions, particularly considering the common mechanism of vascular dysfunction. The vascular damage and disruptions of the microcirculation that occur with FAN can have negative effects not only on bone tissue but also on the broader systemic vascular network. The present study accordingly aimed to clarify the possible common mechanisms of these two different but interrelated diseases by evaluating their pathophysiological and clinical manifestations while drawing attention to the importance of multidisciplinary approaches in treatment strategies. Detailed examination of the epidemiology of both FAN and ED is critical in identifying risk groups and developing proactive strategies for treatment.

## 2. Materials and Methods

### 2.1. Study Design

This study was a retrospective case–control study. The Scientific Research Ethics Committee of the University of Health Sciences Erzurum Faculty of Medicine approved the research (Reference No. 34922). The study was subsequently conducted between January 2020 and December 2023 in the Orthopedics and Traumatology Clinic of the University of Health Sciences Erzurum City Hospital. All analyzed patients were male and had been admitted to outpatient clinics for non-traumatic elective purposes. A total of 92 patients with femur avascular necrosis were evaluated. Fifty patients with complete detailed clinical findings and MRI and X-ray imaging, required for Ficat–Arlet staging, were included in the study. The control group consisted of 50 healthy male individuals, selected from an initial pool of 88 candidates, who were evaluated through clinical examination, plain radiography, and magnetic resonance imaging (MRI) to confirm the absence of femoral avascular necrosis (FAN), classified as Ficat–Arlet stage 0. Individuals with any chronic illness, a history of drug use, prior pelvic surgeries, or neurological conditions associated with erectile dysfunction (ED) were excluded. All 100 people included in the study were married or had a partner and were sexually active and eligible for IIEF scoring.

The study comprised two groups, with one consisting of 50 patients with FAN and the other consisting of 50 healthy individuals in a control group for comparison. The Ficat and Arlet classification is a widely used system for the staging of avascular necrosis of the femoral head based on clinical symptoms and imaging findings. It helps to guide treatment decisions. Patients with FAN were evaluated according to the Ficat–Arlet classification using a combination of plain radiographs, magnetic resonance imaging (MRI), and clinical features. With this classification system, cases of FAN are staged with scores ranging from 1 to 4, with higher scores reflecting more severe FAN. The healthy individuals in the control group also underwent plain radiography, MRI, and clinical examination to confirm stage 0 according to the Ficat–Arlet classification [7].

Both groups were evaluated for ED using the International Index of Erectile Function (IIEF) scale. The International Index of Erectile Function (IIEF) is a widely used, validated questionnaire designed to assess male sexual function. It helps to evaluate erectile dysfunction (ED) severity and treatment outcomes. With this 5-item scale, scores ranging from 1 to 5 are obtained. Patients with no ED complaints receive a score of 5, while those with very severe ED symptoms receive a score of 1 [8].

The Ficat–Arlet staging was performed by an orthopedist and traumatologist, while the IIEF scores were calculated by a urologist.

### 2.2. Patients and Control Group

Non-trauma patients with a minimum of 12 months of orthopedic follow-up were analyzed in this study. All included patients had plain radiographs, MRI scans, and clinical follow-up examinations available in their records. FAN patients whose data were missing and in whom Ficat–Arlet staging could not be performed were excluded. Furthermore, the urological anamnesis of each patient was recorded and the IIEF scale was administered, and then the serum testosterone levels were measured in the morning on an empty stomach. Only individuals with normal serum testosterone levels were included in the analysis. Those with testicular failure that could lead to primary ED, those with multiple sclerosis or Parkinson’s disease, those who had undergone radical pelvic surgery (radical prostatectomy, cystectomy, etc.) or had a history of pelvic radiation, and those using antidepressant drugs or other substances that could cause ED were excluded. The average age of all individuals in both groups was 44.91 years, reflecting the demographic group of young middle age. Individuals under 25 years of age or over 60 were not included in either group. In selecting the upper age limit, we sought to exclude the higher rates of FAN and ED seen in individuals ≥ 60 years of age, as the prevalence of both disorders increases with age. In selecting the lower limit of 25 years [9], we aimed to create homogeneous groups of sexually active individuals with appropriate urological development.

### 2.3. Imaging Protocols

#### 2.3.1. MRI

MRI constitutes the most sensitive imaging technique for the evaluation of FAN, possessing sensitivity of 70–100% and specificity of 90–100%. Since bilateral involvement may be observed in cases of FAN, both hips were included in the field of view of the MRI sequences evaluated in this study. Only patients with confirmed unilateral avascular necrosis were analyzed. Evaluations were based on T1 and T2 sequences. Hyperintense areas such as those affected by early-stage edema, indicated by lower T1 signals, were examined. In T2 sequences, increased intensity between normal bone marrow and ischemic areas was noted and specific evaluations of hip osteonecrosis were performed.

#### 2.3.2. X-Rays

Anteroposterior pelvic radiography was performed for all patients. The sphericity of the femoral head, osteoarthritic and secondary degenerative changes, osteochondral structures, and the presence of subchondral fractures were examined. The possible presence of osteopenia, sclerosis, subchondral cysts, crescent signs, cortical collapse, and degenerative changes, all of which are important in Ficat–Arlet staging, was carefully examined on each plain radiograph.

### 2.4. Data Analysis

To evaluate the data obtained in this study, the NCSS 2020 Statistical Software (NCSS LLC, Kaysville, UT, USA) was utilized for statistical analysis. While quantitative variables are presented using mean, standard deviation, median, minimum, and maximum values, qualitative variables are presented as frequencies and percentages, in line with descriptive statistical methods. Shapiro–Wilk tests and box-plot graphics were utilized to facilitate evaluations of the compliance of all data with a normal distribution. Subsequently, Student *t*-tests were used for quantitative evaluations of normally distributed data from two groups, while Kruskal–Wallis tests were used for comparisons of non-normally distributed variables from three or more groups, and the Dunn test was used to identify the groups from which differences arose. Spearman’s correlation analysis was performed to evaluate possible relationships between variables. Fisher–Freeman–Halton tests were performed for comparisons of qualitative data. All results were evaluated with 95% confidence intervals and a significance level of *p* < 0.05.

## 3. Results

The research was finalized with a total of 100 male participants. Their ages ranged from 29 to 60 years, with a mean age of 44.91 ± 7.37 (Table 1).

According to the FAN staging, stage 1 disease (mild) was detected in 16% (n = 8) of the cases, stage 2 in 40% (n = 20), stage 3 in 32% (n = 16), and stage 4 (severe) in 12.0% (n = 6).

According to the obtained IIEF scores, 4.0% (n = 4) of the participants had severe ED (score of 1), 9% (n = 9) had an ED score of 2, 15% (n = 15) had a score of 3, 25% (n = 25) had a score of 4, and 47% (n = 47) had no ED symptoms (score of 5).

While half of the analyzed individuals were FAN patients (50%, n = 50), 50% (n = 50) constituted the healthy control group (Table 2).

**Figure 1:**
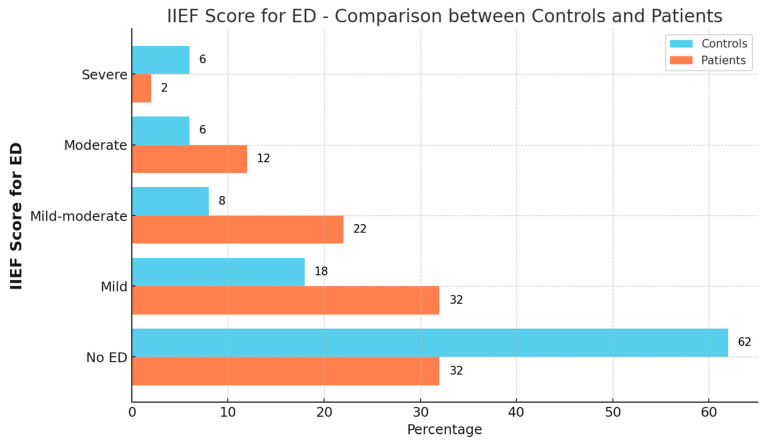
Distribution of IIEF scores by group, reflecting the extent of erectile dysfunction (ED).

The ages of the participants did not differ with statistical significance between the groups (*p* > 0.05). However, the IIEF scores were significantly different between patients with FAN and the healthy control group (*p* = 0.001). The rates of mild to moderate ED, as reflected by scores of 2–4, were higher among FAN patients compared to the control group, while the rate of individuals with no ED symptoms (score of 5) was higher in the control group (Figure 1).

**Figure 2:**
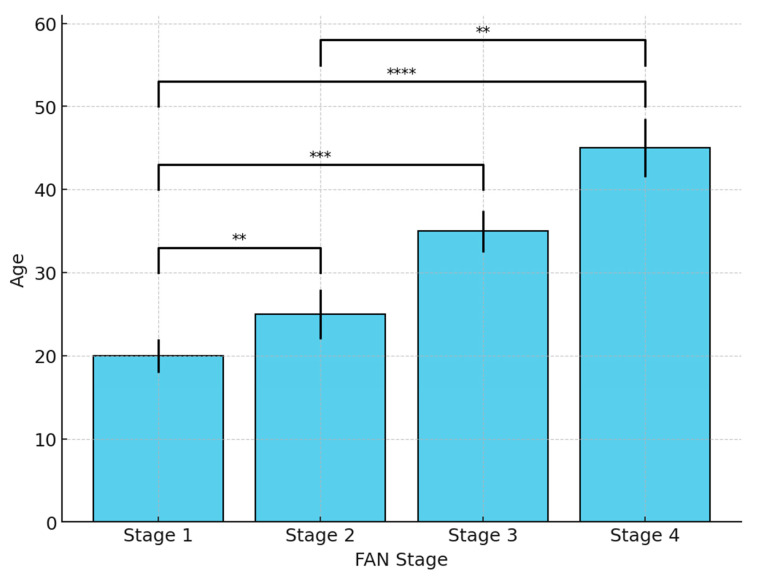
Variations in FAN stage according to age. **, *p* < 0.01; ***, *p* < 0.001; ****, *p* < 0.0001.

The FAN stages were found to vary significantly by age. Specifically, patients with stage 4 FAN according to the Ficat–Arlet classification were statistically significantly older than those with stage 1 or stage 2 FAN (*p* = 0.004 and *p* = 0.001; *p* < 0.01) (Table 3 and Table 4 and Figure 2).

The IIEF scores, reflecting the extent of ED, did not differ significantly according to age (*p* > 0.05).

A statistically significant negative relationship was observed between the FAN stages and IIEF scores. Higher FAN stages, reflecting more severe FAN, correlated with lower IIEF scores, reflecting more severe ED (rho = −0.631; *p* = 0.001).

**Figure 3:**
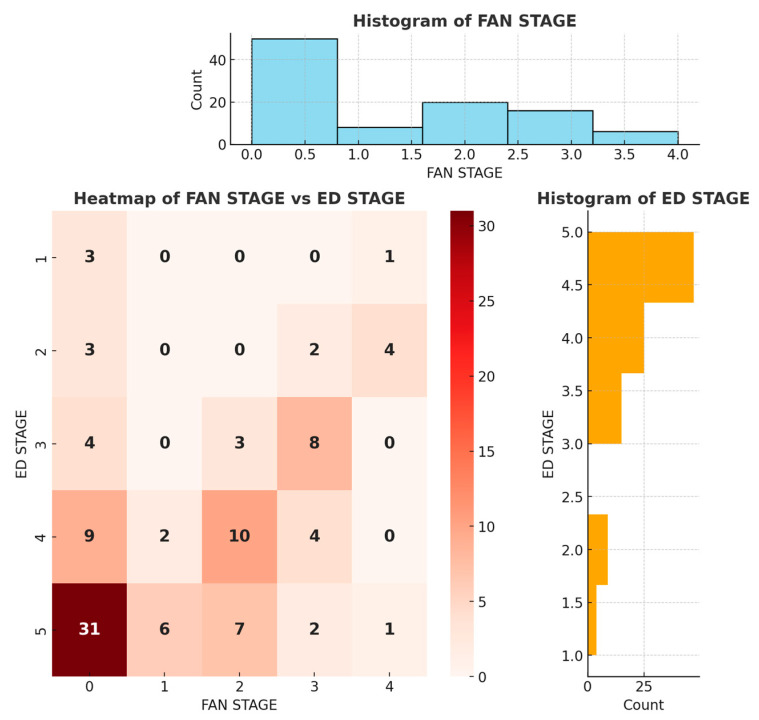
Relationship between FAN stage and IIEF score. While higher FAN scores indicate more severe FAN, lower IIEF scores indicate more severe ED.

The Spearman correlation coefficients (rho, ρ) and *p*-values (*p* < 0.01) indicate a significant and negative association between the FAN stage and IIEF score (erectile function). The negative Spearman correlation indicates that, as the FAN stage increases (i.e., the disease progresses), the severity of ED increases (the IIEF score decreases). *p* < 0.01 indicates that this relationship is statistically highly significant (we can say with 99% confidence that this relationship is not coincidental) (Figure 3).

## 4. Discussion

In this retrospective case–control study, the relationship between FAN and ED was examined. The findings revealed the existence of a statistically significant negative relationship between the FAN stages and IIEF scores (rho = −0.631; *p* = 0.001). The study suggests that, as FAN progresses, the severity of ED increases as a result of the deterioration of systemic vascular health and adverse effects on penile blood flow, but studies involving larger populations are needed.

The similar pathophysiological mechanisms of FAN and ED reflect the ways in which these two diseases are related to each other. Several of the risk factors for the development of FAN, such as corticosteroid use, alcohol consumption, and systemic diseases, disrupt the blood flow to bone tissue and trigger avascular necrosis. The pathogenesis of ED is similarly largely based on vascular dysfunction. The dysregulated modulation of vascular endothelial growth factor (VEGF) and microcirculation disorder constitute common foundations of these diseases [1,2].

In this study, the finding of a higher rate of ED in the patient group versus the control group supports the possibility of FAN causing not only local bone damage but also systemic vascular damage. FAN is a condition that generally occurs as a result of reduced blood flow and it may be associated with systemic vascular complications. In particular, systemic diseases such as diabetes are known to have influential roles in the development of FAN. Lai et al. examined the relationship between diabetes and FAN and stated that vascular complications may be effective in the disease process [10]. It is thought that, particularly in advanced stages of FAN, deterioration in the vascular system may increase the severity of ED by restricting penile blood flow. The present study accordingly highlights the common vascular pathophysiologies of FAN and ED and demonstrates the importance of a multidisciplinary treatment approach.

In our review of the literature, no study was found that directly addressed the relationship between FAN and ED. However, vascular endothelial damage plays a decisive role in the pathophysiologies of both diseases. In line with this common mechanism, extracorporeal shock wave therapy (ESWT) has been reported to provide beneficial results in the treatment of both FAN and ED [11,12]. ESWT is used for both diseases because shock waves trigger neovascularization by increasing VEGF expression within the tissue, thereby improving blood flow [13,14]. The positive effects of ESWT in treating both diseases support the existence of overlapping pathophysiologies between FAN and ED and reveal that evaluating these clinical conditions together is important for the planning of effective treatment strategies.

There is no definitively proven treatment for FAN, neither pharmacologically nor surgically. The literature reports that anticoagulants, bisphosphonates used to inhibit bone destruction, prostaglandin analogs, antiplatelet drugs such as acetyl salicylate, and many other agents are being used for the treatment of FAN [15,16,17,18,19,20,21]. However, no treatment has been shown to offer high levels of evidence, and these treatments have side effects [22]. One previous study involving the administration of sildenafil to rats showed increased levels of VEGF and osteopontin in the early period following ischemic trauma to the femoral head, together with a corresponding increase in the production of new non-calcified (osteoid) bone tissue and matrix, verifying that sildenafil may facilitate the regeneration of bone tissue in cases of ischemic trauma [23]. In another study, vardenafil was shown to have a beneficial impact on bone union and promote the early stages of fracture healing, when vasodilation is more necessary in a setting of increased inflammation [24]. The first-line treatment of ED traditionally entails oral drug therapy with agents such as apomorphine or sildenafil, but two new phosphodiesterase-5 (PDE5) inhibitors, tadalafil and vardenafil, were shown to provide better selectivity [25]. The strong relationship observed in our study between FAN and ED, the positive effects of PDE5 inhibitors on avascular necrosis and bone fracture healing in previous experimental studies, and the successful results previously obtained with PDE5 inhibitors in the treatment of ED signify the potential of these inhibitors for future use in the treatment of patients with FAN. Prospective randomized double-blind and controlled studies are needed to determine the true clinical effectiveness of these compounds, but the present study is important in terms of offering guidance for future research in this area.

When FAN and ED are examined in terms of a common etiology, it is observed that both diseases involve issues related to mineral metabolism. A study suggested that copper metabolism disorders might be responsible for steroid-induced femoral head osteonecrosis [26]. A study conducted by Gonzalez-Reimers et al. [27] confirmed, through experiments on rats, that steroids increase muscle copper, iron, and zinc levels, as well as bone copper levels. Copper (Cu) ions stabilize the expression of hypoxia-inducible factor-1α (HIF-1α) and upregulate the expression of vascular endothelial growth factor (VEGF); high VEGF expression induces neovascularization to further promote bone development [28,29]. Additionally, high copper concentrations have been shown to significantly reduce the proliferation of osteogenic precursor cells and decrease new bone formation [30]. Another study analyzing element concentrations in bone tissue demonstrated a relationship between the free testosterone and magnesium and manganese concentrations, but only in patients with erectile dysfunction. In patients without erectile dysfunction, a correlation was observed between the free testosterone and copper concentrations [31]. Recent studies also indicate that there is a relationship at the elemental level in the pathophysiology of FAN and ED.

Our study has certain limitations. While presenting the fundamental descriptive characteristics between the groups, we had to rely on the accuracy of the available data and the existing patient records. Given the potential relationship among factors such as patients’ height, weight, body mass index, blood pressure, race, occupation, smoking history, alcohol consumption history, substance use history, presence of a partner, corticosteroid use history, history of hip trauma, diabetes, and sickle cell anemia in both ED and FAN [3], it is important that future studies include these variables. These variables were not included in our study because they were either missing or could not be obtained reliably during the retrospective data collection process. Additionally, due to the design of the study, it was not possible to access all variables. Based on the findings of this study, we recommend conducting further research with a prospective design that incorporates a broader range of variables.

However, the present study also has methodological limitations to be acknowledged. Its retrospective design and small sample size may have affected the generalizability of the findings. Future studies comprising larger patient populations, prospective designs, and the inclusion of additional variables such as hormonal profiles and microcirculation parameters would support a more thorough understanding of the relationship between FAN and ED.

In conclusion, this study has demonstrated that the ED severity increases with increasing FAN stages, and the similar vascular pathophysiological mechanisms of these two diseases explain this relationship. Our findings emphasize the crucial value of early diagnosis and multidisciplinary treatment strategies in the management of both diseases. In addition, the early evaluation and management of ED in patients diagnosed with FAN will play an important role in improving their quality of life.

## Figures and Tables

**Table 1:** Descriptive data of all participants.

		n (%)
**Years**	Mean ± SD	44.91 ± 7.37
	Median (range)	45 (29–60)
**FAN stage**	1 (mild)	8 (16.0)
	2	20 (40.0)
	3	16 (32.0)
	4 (severe)	6 (12.0)
**IIEF score**	1 (severe ED)	4 (4.0)
	2	9 (9.0)
	3	15 (15.0)
	4	25 (25.0)
	5 (no ED)	47 (47.0)
**Group**	Patients	50 (50.0)
	Control group	50 (50.0)

**Table 2:** Comparisons of descriptive characteristics by group.

		Patients (n = 50)	Controls (n = 50)	*p*
**Age**	Mean ± SD	45.86 ± 7.54	43.96 ± 7.16	0.199 ^a^
	Median (range)	47 (29–60)	45 (29–57)	
**FAN stage**	1 (mild)	8 (16.0)	0 (0.0)	**-**
	2	20 (40.0)	0 (0.0)	
	3	16 (32.0)	0 (0.0)	
	4 (severe)	6 (12.0)	0 (0.0)	
**IIEF score**	1 (severe ED)	1 (2.0)	3 (6.0)	0.001 ^b,^**
	2 (moderate)	6 (12.0)	3 (6.0)	
	3 (mild–moderate)	11 (22.0)	4 (8.0)	
	4 (mild)	16 (32.0)	9 (18.0)	
	5 (no ED)	16 (32.0)	31 (62.0)	

^a^: Student *t*-test; ^b^: Fisher–Freeman–Halton test; **: *p* < 0.01.

**Table 3:** FAN stages by age.

FAN Stage (n = 50)	Mean ± SD	Median (Range)	Kruskal–Wallis *p*-Value
1 (Mild)	42.38 ± 5.97	42.5 (34–53)	0.001
2	42.65 ± 7.79	42 (29–55)	
3	47.63 ± 4.22	49 (41–53)	
4 (Severe)	56.5 ± 4.09	58 (50–60)	

**Table 4:** IIEF scores by age.

IIEF Score (n = 100)	Mean ± SD	Median (Range)	Kruskal–Wallis *p*-Value	Dunn–Bonferroni *p*-Value
1 (Severe ED)	49.50 ± 8.19	51.5 (38–57)	0.053	-
2 (Moderate ED)	50.89 ± 7.32	51 (41–60)		-
3 (Mild–Moderate ED)	46.93 ± 3.92	47 (41–55)		-
4 (Mild ED)	43.48 ± 7.23	44 (29–55)		-
5 (No ED)	43.49 ± 7.62	43 (32–58)		-

## Data Availability

The raw data supporting the conclusions of this article will be made available by the authors upon request.

## Abbreviations

The following abbreviations are used in this manuscript:

FAN	Femoral head avascular necrosis
ED	Erectile dysfunction
IIEF	International Index of Erectile Function
MRI	Magnetic resonance imaging
VEGF	Vascular endothelial growth factor
ESWT	Extracorporeal shock wave therapy
PDE 5	Phosphodiesterase-5
